# Strategies of oral hygiene care for healthy aging: insights from elderly care in Taiwan

**DOI:** 10.3389/fragi.2026.1811756

**Published:** 2026-05-29

**Authors:** Ming-Kuo Chen, Patrick Pang, Yi-Huang Shih, Ting Liu, Huiwen Zou

**Affiliations:** 1 Chaoyang University of Technology, Taichung, Taiwan; 2 Faculty of Applied Sciences, Macao Polytechnic University, Macau, Macao SAR, China; 3 Center of Teacher Education, Minghsin University of Science and Technology, Xinfeng, Taiwan

**Keywords:** healthy aging, older adults, oral health, tooth, wellbeing

## 1 Introduction

### 1.1 Oral frailty should be incorporated into the core indicator system of healthy aging

In recent years, a growing body of research on oral frailty has indicated that it should not be regarded merely as a localized dental condition, but rather as a potential early marker of functional decline within the broader trajectory of healthy aging. For instance, a cross-sectional study of community-dwelling older adults in the northeastern border regions of China reported a relatively high prevalence of oral frailty. The study further revealed significant associations between oral frailty and advanced age, multimorbidity, risk of malnutrition, depressive symptoms, and decline in activities of daily living (ADL). Collectively, these findings underscore the close relationship between oral frailty and overall functional deterioration, suggesting that oral frailty may be more appropriately conceptualized as a geriatric syndrome characterized by multidimensional vulnerability rather than as an isolated oral health issue (Wang et al., 2024). Therefore, oral frailty should be incorporated into the core indicator system of healthy aging.

### 1.2 Taiwan’s experience may serve as a governance model for oral health in super-aged societies (2010–2026)

Microbes appear in every corner of human life, and microbes affect every aspect of human life. The human oral cavity contains a number of different habitats. Synergy and interaction of variable oral microorganisms help human body against invasion of undesirable stimulation outside. However, imbalance of microbial flora contributes to oral and systemic diseases. In the context of an aging society, the importance of healthcare for older adults has become increasingly prominent. Oral health, as a fundamental basis for maintaining general health and quality of life, represents the first line of defense in geriatric healthcare. With advancing age, older adults often face multiple oral health challenges, including tooth loss, alveolar bone resorption, oral mucosal diseases, declines in chewing and swallowing function, and xerostomia resulting from chronic illnesses or medication side effects. These conditions not only compromise nutritional intake—leading to weight loss, sarcopenia, and immune decline—but are also closely linked to systemic health outcomes. Empirical studies have demonstrated that oral infections and periodontal disease are associated with cardiovascular disease, diabetes, respiratory infections, and dementia (Gao et al., 2018; Nangle et al., 2019; Tonetti and Jepsen, 2013). From a life-course and healthy aging perspective, oral health also plays a crucial role in older adults’ psychological and social wellbeing. Tooth loss and functional limitations may impair speech and alter appearance, thereby reducing self-esteem, social participation, and interpersonal interaction, and contributing to isolation and depressive symptoms (Gerritsen et al., 2010). Conversely, maintaining adequate oral function promotes dietary diversity, improves masticatory efficiency, and enhances social engagement, all of which support overall quality of life, and promot older adults’ healthy aging (Tonetti et al., 2017; Locker and Allen, 2007). Accordingly, oral healthcare for older adults should be conceptualized not merely as a dental issue but as an integral component of comprehensive health management and public health policy in aging societies. Such an approach requires preventive strategies, functional maintenance, and interdisciplinary collaboration among health professionals. This article focuses exclusively on strategies of oral hygiene care for healthy aging, drawing insights from elderly care in Taiwan. Taiwan’s experience may serve as a governance model for oral health in super-aged societies.

## 2 What is healthy aging

Public health faces escalating challenges because worldwide, population aging advances forward. From the perspective of the current context, it is essential to aim for the preservation of a satisfactory oral health condition in the elderly population. For this reason, the United Nations and the World Health Organization (WHO) allocated the years between 2021 and 2030 as the Decade of Healthy Aging with an intent to encourage concerted action to promote longer, healthier lives (Asanza et al., 2025). Every person–in every country in the world–should have the opportunity to live a long and healthy life. Yet, the environments in which we live can favour health or be harmful to it. Environments are highly influential on our behaviour, our exposure to health risks (for example, air pollution or violence), our access to quality health and social care and the opportunities that ageing brings. Healthy ageing is about creating the environments and opportunities that enable people to be and do what they value throughout their lives (World Health Organization, 2026).

Ageing is a dynamic biological process involving interconnected physiological, psychological, and social changes, making the promotion of healthy ageing a global public health priority. The WHO defines healthy ageing as the process of developing and maintaining functional ability that enables wellbeing in older age. The WHO’s Decade of Healthy Aging (2021–2030) outlines four key action areas: changing attitudes toward ageing, creating age-friendly environments, delivering integrated and person-centred care, and ensuring access to long-term care (Garg et al., 2026; Shih, 2022). In addition, existing research has demonstrated that the external environment not only directly influences life-space mobility among community-dwelling older adults, but also exerts an indirect effect through intrinsic capacity. This evidence points to a pathway mechanism linking “environment–intrinsic capacity–functional performance.” Within this conceptual framework, declining oral function may compromise nutritional intake and reduce physiological reserves, thereby weakening intrinsic capacity and ultimately constraining life-space mobility and social participation. It is therefore recommended to construct a conceptual pathway model—such as “oral function–nutrition–sarcopenia–cognition–social participation”—and to explicitly situate oral health within the WHO functional ability framework in order to strengthen theoretical integration and explanatory coherence (Wang et al., 2023; Wang et al., 2026).

## 3 A new trend in elderly care: oral healthcare

In response to the global trend of demographic aging, the United Nations and many countries—such as Japan, Sweden, and France—have established comprehensive research organizations and institutions dedicated to aging studies, strengthening research on issues related to older adults. In 1999, the United Nations designated the International Year of Older Persons, advocating for “a society for all ages” and emphasizing the importance of active aging. In 2002, the WHO introduced the policy framework of active aging, which promotes the enhancement of older adults’ quality of life through three key pillars: health, participation, and security. The WHO further clarified that the concept of active aging evolved progressively from related ideas such as successful aging, productive aging, and healthy aging. Since then, active aging has become a central guiding principle in health policies for older populations worldwide (Ministry of Health and Welfare, 2015; Huang, 2020).

In efforts to enhance the quality of life of older adults, oral healthcare has emerged as a new and significant trend in older adults care. Oral diseases are among the most common health problems affecting older populations. Poor oral health in later life is often associated with higher rates of tooth loss, dental caries, periodontal disease, xerostomia, and oral cancer, all of which adversely affect quality of life. Preventing oral diseases among older adults can reduce national expenditures on medical treatment and long-term care. Moreover, the social, physiological, and psychological impacts brought about by population aging have become major public health concerns worldwide (Ministry of Health and Welfare, 2015; Ministry of the Interior, 2023).

## 4 Strategies for promoting oral health among older adults in Taiwan

Drawing on the publications of the Ministry of Health and Welfare (2015) and the New Taipei City Government (2016), this study proposes four strategies, which are discussed in detail below.

### 4.1 Cultivating healthy lifestyle habits among older adults

Lifestyle-related diseases such as dental caries and periodontal disease are closely associated with daily behavioral patterns. Poor oral health is often linked to inadequate chewing habits, unhealthy dietary patterns, and insufficient daily oral hygiene practices. Therefore, reducing unhealthy lifestyle behaviors among older adults is essential. These behaviors include smoking, alcohol consumption, intake of caffeinated beverages (such as tea, coffee, and cola), chronic stress, sleep deprivation, and habitual mouth breathing. Cultivating healthy lifestyle habits among older adults constitutes an important strategy for promoting oral health. Such efforts not only enhance oral function but also contribute to improvements in overall physical health and wellbeing (Ministry of Health and Welfare, 2015; New Taipei City Government, 2016).

### 4.2 Oral health exercises for older adults

Oral health exercises (Jian-Kou exercises) are structured movements targeting the oral cavity, head and neck region, and hands. Regular daily practice of these exercises can strengthen oral musculature, stimulate salivary secretion, enhance masticatory function, and improve chewing and swallowing abilities. Such improvements may reduce the risk of choking and aspiration, broaden dietary options, promote better nutritional absorption, and ultimately enhance overall health status. Oral frailty refers to a decline in oral function. Early detection of subtle deterioration in oral function, followed by timely intervention through oral health exercises, can effectively restore and enhance oral capacity among older adults (Department of Oral HygieneKaohsiung Medical University, 2012). As oral function improves, older adults are better able to eat independently, which contributes positively to their physical health and overall wellbeing (New Taipei City Government, 2016).

### 4.3 Oral hygiene practices for older adults

Dental and oral care play a vital role in maintaining a healthy life. Numerous reports have demonstrated a strong association between oral health and quality of life among older adults. Consequently, oral hygiene care for the older adults has received increasing attention in recent years. However, older adults who lack self-care capacity are often unable to clean their teeth independently. Furthermore, assisting with oral hygiene can be challenging for caregivers, resulting in poor oral conditions among many dependent older individuals (Department of Oral HygieneKaohsiung Medical University, 2012). Proper oral hygiene is essential for disease prevention and overall wellbeing. Individuals who maintain good oral cleanliness and utilize appropriate occlusal function for adequate mastication may preserve vitality and potentially reduce the risk of cognitive decline. For older adults, effective use of dentures to facilitate proper chewing not only stimulates brain activity but also reduces the number of unhealthy days, contributing to longevity, functional independence, and active aging (Japan Academy of Gnathology and Occlusion, 2015). Moreover, a Japanese study conducted in long-term care institutions found that when caregivers assisted residents with daily toothbrushing and dentists provided professional mechanical cleaning once per month, the incidence of pneumonia decreased by 40% after 1 year (Department of Oral HygieneKaohsiung Medical University, 2012). The above findings underscore the importance of oral hygiene among older adults. Recommended oral healthcare practices include: (1) Proper toothbrushing within 3 minutes after meals and before bedtime. (2) Use of appropriate oral hygiene tools, including soft-bristled, small-headed toothbrushes and dental floss; interdental brushes are recommended for individuals with periodontal disease or larger interdental spaces. (3) Avoidance of unhealthy habits such as smoking, alcohol consumption, and betel nut chewing. (4) Regular oral examinations every 6 months (New Taipei City Government, 2016).

### 4.4 The “333 tooth-brushing rule”

Existing literature on oral health and hygiene, both domestically and internationally, indicates that effective oral hygiene care not only helps prevent the progression of diseases but also contributes significantly to reducing disease incidence, promoting oral health, and improving overall quality of life (Liao and Wang, 2021). Among daily oral health practices, tooth brushing is one of the most fundamental activities performed regularly. However, for older adults, this seemingly simple task may become increasingly challenging due to age-related declines in physical function, reduced hand dexterity, or the presence of chronic diseases (Li and Hu, 2015). Tooth brushing is a crucial method for maintaining oral cleanliness among older adults. Proper brushing helps remove debris, food particles, and dental plaque from the surfaces of teeth. It also provides a gentle massaging effect on the gums, which contributes to improving the oral environment and reducing pathogenic factors, thereby enhancing older adults’ resistance to disease. Therefore, oral health education programs for older adults may promote the concept of the “333 tooth-brushing rule.” This guideline recommends brushing after three daily meals and before bedtime, brushing within 3 minutes after eating, and brushing for at least 3 minutes each time. Such a simple and memorable guideline can effectively encourage consistent oral hygiene practices among older adults (New Taipei City Government, 2016).

## 5 Reflections

If the discussion is to be further extended from an institutional and theoretical perspective, Taiwan has officially entered a super-aged society. This demographic transition represents not merely a statistical shift in population structure, but a fundamental transformation in health governance and public policy design. Within this context, oral health should be incorporated into the broader policy framework of healthy aging rather than being confined to the domain of individual hygiene practices or clinical dental treatment. According to the WHO (World Health Organization, 2015), healthy aging centers on the maintenance and promotion of functional ability, which is shaped by the dynamic interaction between intrinsic capacity and environmental support. From this perspective, declining oral function—if it compromises nutritional intake, physiological reserves, and cognitive performance—extends beyond a dental issue and directly affects intrinsic capacity and functional performance. First, at the level of the National Health Insurance (NHI) system, Taiwan’s single-payer model ensures financial accessibility; however, dental reimbursement remains largely treatment-oriented, with limited emphasis on preventive and function-maintenance services for older adults. In alignment with the healthy aging framework, policy design should shift from a disease-treatment model toward a function-preservation model, incorporating oral functional assessment and preventive interventions as core service components (Patel and Gallagher, 2024; Tonetti et al., 2017). Second, within the Long-Term Care (LTC 2.0) policy framework, oral health has not yet been fully institutionalized into interdisciplinary care planning. Empirical studies indicate significant associations between oral health, malnutrition, sarcopenia, and cognitive decline (Locker and Allen, 2007; Gao et al., 2018). Failure to integrate oral assessment into long-term care processes may result in overlooking a modifiable risk factor for functional deterioration. Therefore, interdisciplinary collaboration among dentists, nurses, dietitians, and case managers should become a central component of system design. Third, at the level of community health networks, Taiwan possesses a well-developed primary care and public health infrastructure. Nevertheless, many oral health initiatives remain focused on screening and health promotion campaigns, with limited long-term monitoring and integration of functional indicators. From a life-course perspective, oral health should be conceptualized as a determinant of social participation and life-space mobility rather than as an isolated clinical concern (World Health Organization, 2015).

In summary, healthy ageing means more than disease free but the maintenance of functional ability in older adults. The World Health Organization proposed a 10-year action plan in Decade of Healthy Ageing 2021–2030 to encourage countries to develop and implement policy and supportive programmes in promoting healthy ageing and improving the quality of life in older adults. Older adults will be the centre in this plan with all the stakeholders including policymakers, national professional associations, academics and all healthcare professionals working together to improve the lives of older adults, their families and communities in the coming decade (Chan, Chu, Ogawa, Lai, 2024). Theoretical integration may be strengthened by constructing a conceptual pathway model—such as “oral function–nutrition–sarcopenia–cognition–social participation”—and embedding it within the WHO functional ability framework. This approach aligns with the healthy aging paradigm’s emphasis on maintaining functional ability across the life course and highlights the multidimensional interactions between physiological, nutritional, and social determinants of health (Cruz-Jentoft et al., 2019; World Health Organization, 2015). Such integration would not only reinforce the theoretical coherence of the manuscript but also deepen its discussion of institutional arrangements and public health governance in aging societies. Taiwan’s experience may serve as a governance model for oral health in super-aged societies.

## 6 Discussion

Education is a fundamental pillar of sustainable development, providing individuals with knowledge, skills, and opportunities for personal growth and socioeconomic advancement (Chen and Wu, 2024; Huang et al., 2024; Liu et al., 2024; Shih, 2026; Wang and Shih, 2022; 2023; Wu, 2022; Zou et al., 2025). This also includes providing oral hygiene education for older adults. In Taiwan, as the post-war baby boom generation gradually enters old age, coupled with a continued decline in birth rates, the pace of population aging in Taiwan has accelerated significantly. Population aging has become one of the most critical issues affecting social development, not only reshaping demographic structures but also profoundly influencing social composition and the allocation of national labor resources. According to the WHO, a country is classified as an “aging society” when individuals aged 65 and over account for 7% or more of the total population. Projections further indicate that by 2026, this proportion will exceed 20%, reaching the threshold of a “hyper-aged society.” (Liu et al., 2024; Ministry of Health and Welfare, 2015; Huang, 2020; Shih et al., 2022; Shih, 2024; Wei and Yang, 2010). As society ages rapidly, the increase in an ageing population has given rise to ‘ageism’, with common preconceptions of older people being a ‘burden’. The relationship between health status and quality of life for older adults has received increasing attention. Scholars generally agree that the impact of disease should be considered when assessing overall health. Tooth loss, which is often caused by oral diseases, is likely to have a significant negative effect on Oral Health-Related Quality of Life (OHRQoL) (Gerritsen et al., 2010). Therefore, this paper aims to explore strategies for promoting oral health among older adults in Taiwan.

From the perspective of healthy aging, maintaining functional ability requires sustained attention to intrinsic capacity and environmental support (World Health Organization, 2015). In this regard, oral health plays a foundational role in preserving nutritional status, physical resilience, and social participation among older adults (Tonetti et al., 2017). First, regarding urban–rural disparities, although Taiwan’s National Health Insurance (NHI) system ensures financial accessibility, dental workforce distribution remains uneven. Metropolitan areas have a higher dentist-to-population ratio, whereas rural and offshore regions face relative shortages. Structural inequities in healthcare access may hinder early detection and intervention for oral frailty, thereby exacerbating functional decline among vulnerable older adults (Gao et al., 2018). Second, Taiwan became an aged society in March 2018, and is on course to become a hyper-aged society by 2026. To adapt to the enormous rising need for long-term care and to mitigate the burden of family caregivers, the government launched the 10-year Long-term Care Plan 2.0 in 2017. The plan actively expands home, community and residential care services; promotes a new subsidy schedule to support customized care plans; and strengthens comprehensive community care systems. These initiatives create a quality, affordable and accessible long-term care system that meets the diverse needs of the people of Taiwan (Ministry of Health and Welfare, 2026). Within the Long-Term Care 2.0 framework, oral health has not yet been fully institutionalized into standardized assessment and interdisciplinary care planning. Evidence suggests that oral dysfunction is associated with malnutrition, sarcopenia, and cognitive decline, all of which contribute to reduced intrinsic capacity and functional performance (Locker and Allen, 2007; Tonetti and Jepsen, 2013). Functionally impaired older adults often depend on caregivers for daily oral hygiene; however, caregiving staff frequently lack systematic oral health training. Without structured education programs and cross-professional collaboration, oral care may be deprioritized, delaying preventive action. Third, escalating fiscal and workforce pressures accompany population aging. Early identification and management of oral frailty may represent a cost-effective preventive strategy by mitigating downstream disability and healthcare expenditures (Duncan et al., 2007; Patel and Gallagher, 2024). Embedding oral function assessment within community-based integrated health management systems would align with the WHO healthy aging framework, which emphasizes maintaining functional ability across the life course (World Health Organization, 2015). From a research and policy development perspective, several priorities merit consideration: (1) Longitudinal intervention studies examining whether improvements in oral function can delay declines in intrinsic capacity and life-space mobility (Clements and Sarama, 2020; World Health Organization, 2015). (2) Community-based oral frailty screening mechanisms, integrated into public health centers and aging-friendly community networks. (3) Interdisciplinary care models linking dentistry, nursing, nutrition, and long-term care case management. (4) Comparative institutional research, particularly between Taiwan and Japan, to evaluate outreach dental services and home-based dental care models in super-aged societies. Overall, elderly individuals have a relatively high incidence of oral frailty. Factors influencing oral frailty in elderly individuals include advanced age, female sex, low oral health score, few remaining natural teeth and denture use, chronic disease history, physical frailty, poor motor function, living alone and eating alone, depression, smoking and poor sleep quality. Early screening and intervention should be carried out to reduce the impact of adverse outcomes on patients. Integrating the conceptual linkage between oral frailty, intrinsic capacity, and life-space mobility into Taiwan’s long-term care and public health policy framework would elevate the discussion from individual behavioral practices to systemic governance and health strategy. Such theoretical integration would strengthen both the scholarly rigor and international relevance of the analysis (Lu et al., 2025; Wu et al., 2025).
